# Prognostic Value of Germline Copy Number Variants and Environmental Exposures in Non-small Cell Lung Cancer

**DOI:** 10.3389/fgene.2021.681857

**Published:** 2021-06-11

**Authors:** Shizhen Chen, Liming Lu, Jianfeng Xian, Changhong Shi, Jinbin Chen, Boqi Rao, Fuman Qiu, Jiachun Lu, Lei Yang

**Affiliations:** ^1^The State Key Laboratory of Respiratory Disease, Institute of Public Health, Guangzhou Medical University, Guangzhou, China; ^2^The State Key Laboratory of Respiratory Disease, Guangzhou Institute of Respiratory Diseases, The First Affiliated Hospital of Guangzhou Medical University, Guangzhou, China

**Keywords:** germline copy number variant, non-small cell lung cancer, overall survival, gene-environment interaction, nomogram

## Abstract

Germline copy number variant (gCNV) has been studied as a genetic determinant for prognosis of several types of cancer, but little is known about how it affects non-small cell lung cancer (NSCLC) prognosis. We aimed to develop a prognostic nomogram for NSCLC based on gCNVs. Promising gCNVs that are associated with overall survival (OS) of NSCLC were sorted by analyzing the TCGA data and were validated in a small Chinese population. Then the successfully verified gCNVs were determined in a training cohort (*n* = 570) to develop a prognostic nomogram, and in a validation cohort (*n* = 465) to validate the nomogram. Thirty-five OS-related gCNVs were sorted and were reduced to 15 predictors by the Lasso regression analysis. Of them, only CNVR395.1 and CNVR2239.1 were confirmed to be associated with OS of NSCLC in the Chinese population. High polygenic risk score (PRS), which was calculated by the hazard effects of CNVR395.1 and CNVR2239.1, exerted a significantly higher death rate in the training cohort (HR = 1.41, 95%CI: 1.16–1.74) and validation cohort (HR = 1.42, 95%CI: 1.13–1.77) than low PRS. The nomogram incorporating PRS and surrounding factors, achieved admissible concordance indexes of 0.678 (95%CI: 0.664–0.693) and 0.686 (95%CI: 0.670–0.702) in predicting OS in the training and validation cohorts, respectively, and had well-fitted calibration curves. Moreover, an interaction between PRS and asbestos exposure was observed on affecting OS (*P*_*interaction*_ = 0.042). Our analysis developed a nomogram that achieved an admissible prediction of NSCLC survival, which would be beneficial to the personalized intervention of NSCLC.

## Highlights

We for the first time demonstrated the clinical significance of germline copy number variants at the genome-wide level on predicting the overall survival of individuals affected by NSCLC. A nomogram incorporating germline copy number variants and environmental exposures achieved an admissible concordance index in predicting the overall survival of NSCLC.

## Introduction

Lung cancer has been the leading cause of cancer-related death in China, which has resulted in an estimated 690,567 deaths in 2018 (Global Cancer Observatory: Cancer Today^1^, Accessed: 2 Dec 2020). Unlike the declining trend in the United States, burden of lung cancer has soared in China, as age-standardized lung cancer mortality and years of life lost had increased by 28.2 and 12.6%, respectively, in the past 30 years (Zhou et al., 2019). Unfortunately, newer therapies such as biomarker-targeted treatments have achieved incremental benefits in treating lung cancer over the last decade, but the 5-year survival rate was still low (<21%) (Lu et al., 2019). The effectiveness of targeted treatments varies significantly among different individuals. Since the treatment outcome depends on a combined effect of environmental exposures and genetic variants, we still need to make great efforts to discover determinants that play roles in modulating lung cancer survival.

Germline copy number variant (gCNV) is one of the major heritable variations, which has been recognized as a contributor to cancer risk and unfavorable prognosis (Kuiper et al., 2010; Sapkota et al., 2016; Kumaran et al., 2017; Hu et al., 2018). However, few studies have investigated the effects of gCNVs on lung cancer survival (Liu et al., 2012; Yang et al., 2015b), because most studies focused on somatic CNVs (Yang et al., 2011; Araujo et al., 2015; Zhang et al., 2019; Xu et al., 2020). Since gCNV is more reliable than somatic CNV, it can be a better biomarker than somatic CNV, which differed at the single-cell level and was variable in response to external stimuli (Huang et al., 2011; Voet et al., 2013; Zare et al., 2017). Therefore, association analysis between lung cancer and gCNVs is warranted. Furthermore, considering the effect of genetic variants is modified by environmental exposures (Mbemi et al., 2020), interaction between environmental factors and gCNVs is also an important topic of study. In the current study, we sorted candidate survival-related gCNVs at the genome-wide level using the non-small cell lung cancer (NSCLC) cases from the Cancer Genome Atlas (TCGA) database, and evaluated associations of promising gCNVs with NSCLC overall survival (OS) in a large cohort of 1,248 NSCLC cases in southern Chinese. We also constructed a nomogram for predicting NSCLC survival based on the gCNVs and surrounding factors, which would be beneficial to personalized intervention of NSCLC.

## Materials and Methods

### TCGA Genome-Wide gCNV Data Analysis and Their Associations With NSCLC OS

The raw gCNV data of each TCGA lung adenocarcinoma (LUAD) and lung squamous cell carcinoma (LSCC) case, which records as blood-derived with the “sample_type” code as 10, was manually downloaded from https://portal.gdc.cancer.gov/(Access date: June 1, 2019). Each raw individual file includes information about gCNV site, chromosome region, number of probes from the Affymetrix SNP 6.0 assay, segment mean. Copy number of each gCNV site was calculated by the segment_mean using the formula: copy number = 2 × 2^segment mean^. An example for the individual with TCGA ID as TCGA-4B-A93V is presented as Supplementary Data. Since the gCNV sites named in the raw dataset present no identifiable feature concerning any published data or public gCNV database, and they also differ among each individual, we recompiled the copy number data into identifiable gCNV sites that were previously reported for East Asian (Park et al., 2010), by matching the chromosome location with self-written code in R software. The R code is summarized in Supplementary Methods.

### Study Population

We performed a long-term retrospective cohort study between 2006 and 2019 in two NSCLC-affected southern Chinese populations. All subjects were followed up semiannually by telephone from the time of enrollment until death or the last scheduled follow-up. OS time was calculated from the date when patients were firstly diagnosed to the date of last follow-up if they were alive or death day. The inclusion criteria were: (1) Pathologically confirmed primary NSCLC, (2) No treatment prior to blood donation, (3) unrelated-ethnic Han Chinese. Patients who had a history of other cancers, or had incomplete pathological features or environmental exposure information, or lost to follow-up were excluded. There was no difference in distribution of demographics between studied subjects and excluded ones. 213 eligible patients who were recruited from 2015 to 2019 in Guangzhou city were used for validating OS-related gCNVs of NSCLC that were identified by analyzing the TCGA data. Then 570 NSCLC patients who were enrolled in Guangzhou city were included into a training cohort for development of a prognostic nomogram, and those who were enrolled in Suzhou city were entered into a validation cohort to validate the nomogram. Participants in the two cohorts have been previously described (Yang et al.,2015a,b). The detailed definitions of selected variables could also be found in our previously published studies (Yang et al., 2014, 2015a,b). The study was approved by the institutional review boards of Guangzhou Medical University and Soochow University.

### Determination of Copy Number of Promising gCNVs

Genomic DNA was isolated from peripheral venous blood as described previously (Liu et al., 2012). The Accucopy assay was first performed to determine the copies of promising gCNVs that showed significant associations with OS of TCGA NSCLC patients by a commercial biotechnology company (Genesky Bio-Tech Co., Ltd., Shanghai, China; Supplementary Figure 1a) (Du et al., 2012). Then the standard Taqman copy number assay was used to determine the copy number of two gCNVs (i.e., CNVR395.1 and CNVR2239.1) following the instruction of Applied BioSystems (Thermo Fisher Scientific, MA, United States)^2^. Two experimental probes (i.e., cat# Hs07536445_cn for CNVR395.1 and cat# Hs03282916_cn for CNVR2239.1) were used. PCR was run on an ABI 7900HT fast real-time PCR System (Thermo Fisher Scientific) using the 384–well plate. Three DNA samples with copy number as 2-copy for each gCNV, which were determined by the Accucopy assay, were used as standard samples in every test. Each sample was tested twice. The copy number was automatically determined by the software CopyCaller 2.0 (Thermo Fisher Scientific^3^; Supplementary Figure 1b).

### Agarose Gel Electrophoresis

To visually confirm the loss of CNVR395.1 and CNVR2239.1 in cases, we performed PCR to amplify several DNA fragments that reside in or around the CNVR395.1 and CNVR2239.1. The primer sequences are listed in the Supplementary Table 1.

### Statistical Analysis

After mining the copy number and corresponding clinical data of each TCGA subject, the univariate Cox regression model was used to determine the OS-related gCNVs (Bradburn et al., 2003). Then a least absolute shrinkage and selection operator (Lasso) regression model was used to select the most prognostic gCNVs from all OS-related gCNVs (Tibshirani, 1997). The log-rank test, univariate or multivariate Cox regression model were used to evaluate associations of these gCNVs and surrounding factors with OS in our Chinese cohorts, with estimation of hazard ratio (HR) and 95% confidence intervals (CI) (Bradburn et al., 2003). The polygenic risk score (PRS) was then calculated by sum of an individual’s risk copy numbers, weighted by HR (Blechter et al., 2021). The nomogram was based on proportionally converting each Cox regression coefficients to a 0- to 100-point scale (Zhang and Kattan, 2017). Predictive performance of the nomogram was measured by Harrell’s C-index and calibration with 100 bootstrap samples (Steyerberg and Vergouwe, 2014). For validation of the nomogram, the total points of each patient in the validation set were calculated according to the established nomogram, then Cox regression was performed using the total point as a factor, and finally, the Harrell’s C-index and calibration were analyzed (Pencina and D’Agostino, 2004). The multivariate Cox model was also used for multiplicative interaction analysis. All tests were two-sided and evaluated by the Stata software version 16.0 (StataCorp, 2021) or R software version 4.0.1 (R Core Team, 2005). *P* < 0.05 was considered to be statistically significant.

## Results

**FIGURE 1:**
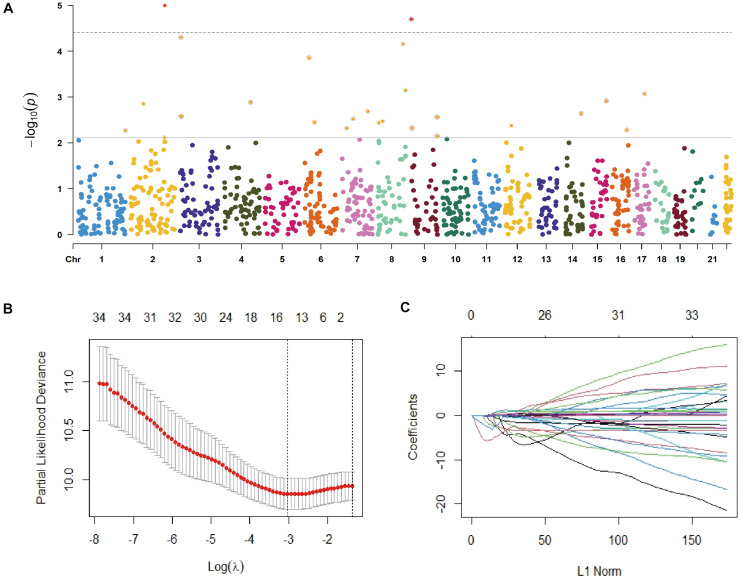
Selection of gCNVs with potentially prognostic value on OS of NSCLC. **(A)** Scatter plot of *P* values in –log10 scale from the univariate Cox model analysis on association between the genome-wide gCNVs and NSCLC OS in the TCGA NSCLC patients. **(B,C)** Selection of predictive gCNVs using the Lasso regression model. Tuning parameter (λ) selection in the Lasso model *via* minimum criteria **(B)** and Lasso coefficient profiles of the 35 OS-related gCNVs **(C)**.

A total of 200 NSCLC cases with available gCNVs data from the TCGA database were analyzed and 35 gCNVs were identified to be significantly associated with OS of NSCLC patients (Figure 1A and Supplementary Table 2). Subsequently, the 35 gCNVs were reduced to 15 potential OS predictors including CNVR_563.1, CNVR_642.1, CNVR_1956.1, CNVR_3560.1, CNVR_431.1, CNVR_2185.1, CNVR_1866.1, CNVR_1837.1, CNVR_2748.1, CNVR_2186.1, CNVR_560.1, CNVR_2239.1, CNVR_395.1, CNVR_564.1, CNVR_2703.1, with non-zero coefficients in the Lasso regression model (Figures 1B,C). These 15 gCNVs were submitted to the Accucopy assay and the CNVR_395.1 and CNVR_2239.1 were sorted to be significantly associated with OS of NSCLC in the 213 cases of Chinese (Supplementary Table 3).

The AGE assay confirmed the copy number loss of CNVR_395.1 and CNVR_2239.1 in population. As shown in Supplementary Figure 2a, no PCR band was produced for primers (P2, P4) to target sequences residing in the CNVR_395.1 in 0-copy samples, but visible PCR products were found for primers to target region upstream of (P1, P3) or downstream (P5) of the gCNV. In contrast, PCR bands were observed for all primers in the 2-copy sample. Interestingly, no band was found not only for the primers (P7) that target sequences residing in the CNVR2239.1, but also for the primers that target sequences upstream of (P6) or downstream of (P8) the gCNV in the 0-copy samples (Supplementary Figure 2b). Since visible bands were found for all primers in the 2-copy sample, this result demonstrates that the chromosomal region of CNVR2239.1 is much larger than reported (Park et al., 2010).

**FIGURE 2:**
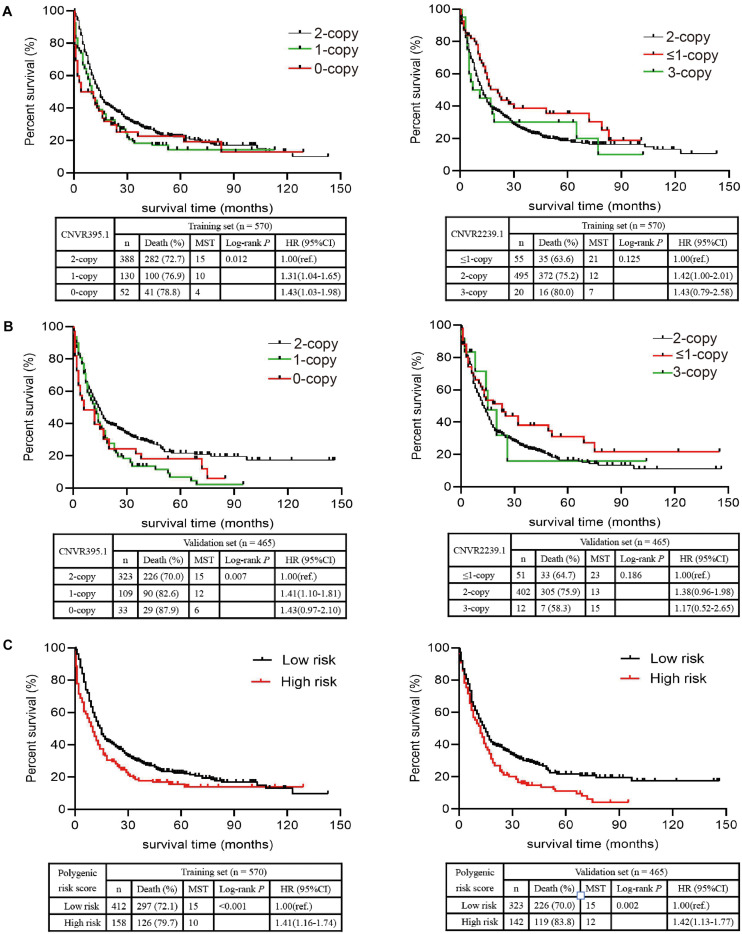
Associations of the CNVR395.1, CNVR2239.1, and PRS with NSCLC OS in Chinese. **(A,B)** The Kaplan–Meier plot was used to visualize the survival probabilities for the CNVR395.1 and CNVR2239.1 in the training cohort **(A)** and the validation cohort **(B)**. **(C)** The Kaplan–Meier plot for PRS.

As shown in Figure 2A, the median survival time (MST) of individuals who carried 1-copy (10 months) and 0-copy (4 months) of CNVR395.1 was significantly shorter than those with 2-copy (15 months, log-rank test: *P* = 0.012). Similarly, cancer-affected individuals with 1-copy and 0-copy had an increased hazard of death (1-copy: HR = 1.31, 95% CI: 1.04–1.65; 0-copy: HR = 1.43, 95% CI: 1.03–1.98). For CNVR2239.1, since there were only 8 cases carrying 0-copy, we combined 0-copy and 1-copy as ≤ 1-copy. There was a growing tendency in MST along with the copy number of CNVR2239.1 increases, but this did not achieve the statistical significance as the log-rank test shown (*P* = 0.125). However, the Cox model revealed that 2-copy of CNVR2239.1 was significantly associated with increased death rate of NSCLC when compared to ≤ 1-copy (HR = 1.42, 95% CI: 1.00–2.01). Moreover, 3-copy conferred a non-significant increase in death rate in comparison with ≤ 1-copy (HR = 1.43, 95% CI: 0.79–2.58), which might be due to the limited sample size. These findings were confirmed in the validation set (Figure 2B). Patients with 0-copy (6 months) and 1-copy (12 months) of CNVR395.1 exhibited a shorter MST and poorer survival rate (1-copy: HR = 1.41, 95% CI: 1.10–1.81; 0-copy: HR = 1.43, 95% CI: 0.97–2.10) than those with 2-copy. Individuals carrying 2-copy of CNVR2239.1 also had a poorer survival rate than those with ≤ 1-copy, with a borderline significance (HR = 1.38, 95% CI: 0.96–1.98). We aggregated the effects of CNVR395.1 and CNVR2239.1 using PRS. Compared to the low PRS, high PRS exhibited substantially reduced MST and increased death rate in both the training set (10 vs. 15 months, *P* < 0.001; HR = 1.41, 95% CI: 1.16–1.74) and the validation set (12 vs. 15 months, *P* = 0.002; HR = 1.42, 95% CI: 1.13–1.77; Figure 2C).

The associations between environmental factors and OS of NSCLC patients are presented in Table 1. In the training set, we observed significantly shorter MST and higher death rate of NSCLC in patients greater than 70 years old than those less than 55 years old (MST: 8 vs. 17 months; HR = 1.61, 95% CI: 1.25–2.07), in patients with pre-existing tuberculosis (TB) than those without (9 vs. 14 months; HR = 1.51, 95% CI: 1.09–2.09), in heavy smokers (>50 pack-years) than light or non-smokers (<5 pack-years) (8 vs. 17 months; HR = 1.66, 95% CI: 1.22–2.25), in patients with asbestos exposure than those without (3 vs. 13 months; HR = 1.61, 95% CI: 1.11–2.33), and in patients with general or bad housing ventilation that those with good condition (11 vs. 17 months; HR = 1.41, 95% CI: 1.14–1.72). However, no significant association with NSCLC survival was observed for sex, pre-existing chronic bronchitis, pre-existing pulmonary emphysema, family history of cancer, family history of lung cancer, arsenic exposure, paint exposure, metallic toxicant exposure, and kitchen ventilator. In the validation set, old age, pre-existing TB, heavy smoke and asbestos exposure were confirmed to be significantly associated with NSCLC OS.

**TABLE 1:** Analysis of the effects of NSCLC-affected individuals’ characteristics and clinical features on NSCLC OS.

Variables	Training cohort (*n* = 570)					Validation cohort (*n* = 465)				
	*n*	Death (%)	MST (m)	*P* value *^*a*^*	HR *^*b*^*	*n*	Death (%)	MST (m)	*P* value*^a^*	HR (95%CI) *^*b*^*
**Polygenic risk score**										
**Sex**										
Male	409	310 (75.8)	12	0.118	1.00(ref.)	319	244 (76.5)	12	0.022	1.00(ref.)
Female	161	113 (70.2)	16		0.85(0.68–1.05)	146	101 (69.2)	16		0.77(0.61–0.97)
**Age (years)**										
<55	179	128 (71.5)	17	< 0.001	1.00(ref.)	126	87 (69.0)	15	< 0.001	1.00(ref.)
55–70	246	179 (72.8)	14		1.13(0.90–1.41)	203	144 (70.9)	15		0.97(0.74–1.26)
>70	145	116 (80.0)	8		1.61(1.25–2.07)	136	114 (83.8)	7		1.69(1.28–2.24)
**Pre-existing TB**										
No	521	383 (73.5)	14	0.011	1.00(ref.)	430	316 (73.5)	14	0.031	1.00(ref.)
Yes	49	40 (81.6)	9		1.51(1.09–2.09)	35	29 (82.9)	5		1.50(1.03–2.20)
**Pre-existing CB**										
No	532	396 (74.4)	13	0.526	1.00(ref.)	431	319 (74.0)	14	0.506	1.00(ref.)
Yes	38	27 (71.1)	12		0.88(0.60–1.31)	34	26 (76.5)	10		1.14(0.77–1.70)
**Pre-existing PE**										
No	549	408 (74.3)	13	0.697	1.00(ref.)	441	327 (74.1)	14	0.281	1.00(ref.)
Yes	21	15 (71.4)	12		0.90(0.54–1.52)	24	18 (75.0)	8		1.29(0.80–2.08)
**Family history of cancer**										
No	525	390 (74.3)	13	0.292	1.00(ref.)	431	322 (74.7)	13	0.623	1.00(ref.)
Yes	45	33 (73.3)	24		0.83(0.58–1.18)	34	23 (67.6)	14		0.90(0.59–1.38)
**Family history of lung cancer**										
No	549	409 (74.5)	13	0.111	1.00(ref.)	455	338 (74.3)	13	0.602	1.00(ref.)
Yes	21	14 (66.7)	29		0.66(0.38–1.12)	10	7 (70.0)	12		0.82(0.39–1.74)
**Pack-year smoked**										
<5	256	179 (69.9)	17	0.002	1.00(ref.)	218	156 (71.6)	15	0.081	1.00(ref.)
5–20	79	57 (72.2)	12		1.28(0.95–1.73)	60	43 (71.7)	16		0.96(0.68–1.34)
20–50	166	132 (79.5)	11		1.38(1.11–1.73)	128	97 (75.8)	11		1.19(0.92–1.54)
>50	69	55 (79.7)	8		1.66(1.22–2.25)	59	49 (83.1)	12		1.44(1.04–1.99)
**Arsenic exposure**										
No	565	418 (74.0)	13	0.557	1.00(ref.)	459	340 (74.1)	14	0.468	1.00(ref.)
Yes	5	5 (100.0)	15		1.29(0.54–3.13)	6	5 (83.3)	6		1.37(0.57–3.33)
**Asbestos exposure**										
No	532	393 (73.9)	13	0.010	1.00(ref.)	440	324 (73.6)	14	0.040	1.00(ref.)
Yes	38	30 (78.9)	3		1.61(1.11–2.33)	25	21 (84.0)	7		1.94(1.25–3.02)
**Paint exposure**										
No	480	362 (75.4)	13	0.207	1.00(ref.)	388	292 (75.3)	13	0.246	1.00(ref.)
Yes	90	61 (67.8)	16		0.84(0.64–1.11)	77	53 (68.8)	17		0.84(0.63–1.13)
**Metallic toxicant exposure**										
No	549	410 (74.7)	13	0.694	1.00(ref.)	442	330 (74.7)	13	0.708	1.00(ref.)
Yes	21	13 (61.9)	12		0.90(0.52–1.56)	23	15 (65.2)	17		0.91(0.54–1.52)
**Housing ventilation**										
Well	179	121 (67.6)	17	0.001	1.00(ref.)	165	119 (72.1)	14	0.256	1.00(ref.)
General/Bad	391	302 (77.2)	11		1.41(1.14–1.72)	300	226 (75.3)	12		1.14(0.91–1.41)
**Kitchen ventilator**										
No	72	50 (69.4)	13	0.397	1.00(ref.)	80	54	14	0.173	1.00(ref.)
Yes	498	373 (74.9)	13		1.22(0.91–1.63)	385	291	13		1.22(0.91–1.63)
**Clinical stages**										
I/II	111	50 (45.0)	72	< 0.001	1.00(ref.)	102	44 (43.1)	54	< 0.001	1.00(ref.)
III	157	126 (80.3)	12		3.29(2.36–4.59)	123	100 (81.3)	14		3.04(2.12–4.34)
IV	302	247 (81.8)	9		3.61(2.65–4.91)	240	201 (83.8)	8		4.16(2.99–5.80)

*MST (m), median survival time (months); HR, hazard ratio; TB, tuberculosis; CB, chronic bronchitis; PE, pulmonary emphysema.*

**^*a*^P* value from the Log-rank test.*

**^*b*^*HR from a Cox regression test.*

**FIGURE 3:**
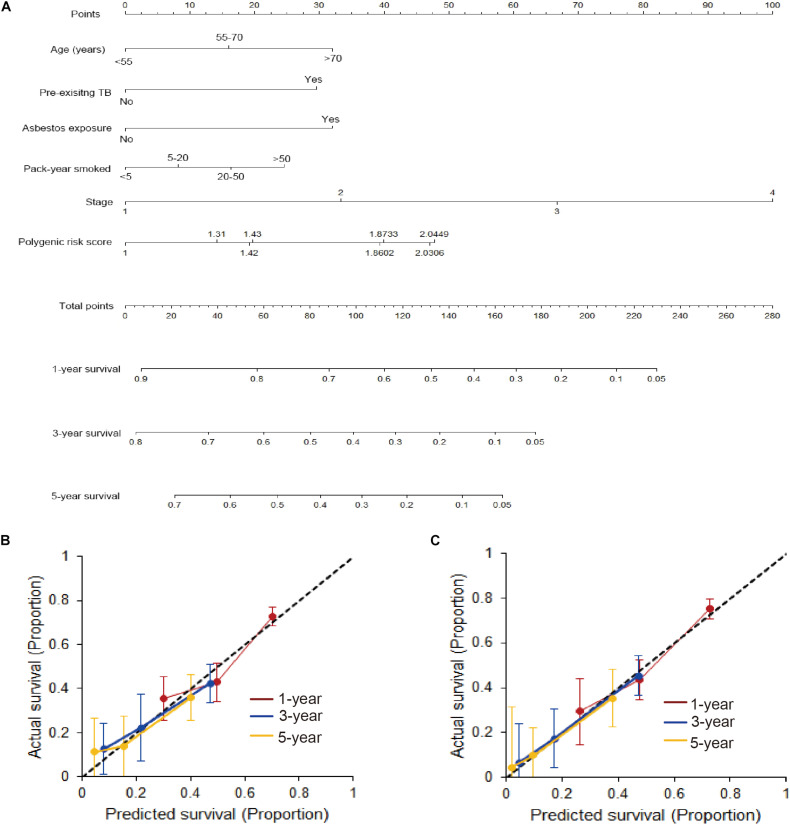
Developed nomogram based on gCNVs and surrounding factors. **(A)** A nomogram was developed in the training cohort with PRS, age, pre-existing TB, asbestos exposure, pack-year smoked and stages incorporated. **(B)** Calibration curve of the nomogram in the training cohort. **(C)** Calibration curve of the nomogram in the validation cohort.

Furthermore, a nomogram that incorporated above significantly prognostic factors was established in the training set (Figure 3A). The calibration plots presented a commendable agreement in both the training and validation cohorts between the actual survival rate and nomogram-predicted survival rate of 1-, 3-, and 5-year (Figures 3B,C). Also, the Harrell’s C-index for the OS model was 0.678 (95%CI = 0.664–0.693) in the training set and 0.686 (95%CI = 0.670–0.702) in the validation set, showing a noticeable value on predicting prognosis of lung cancer.

We combined the training and validation cohorts for the stratification analysis to increase the study power. The multivariate Cox model showed that PRS remained to be significantly associated with NSCLC OS (Supplementary Table 4). We found a multiplicative interaction between PRS and asbestos exposure on affecting NSCLC OS (HR*_*interaction*_* = 1.61, 95% CI: 1.02–2.56; P*_*interaction*_* = 0.042; Table 2). The hazard of death associated with PRS differed when stratified by asbestos exposure, with a stronger association among individuals affected by asbestos exposure (HR = 2.04, 95% CI: 1.32–3.17) compared to those without asbestos exposure (HR = 1.34, 95% CI: 1.14–1.58). The interaction remained unchanged in the multivariate Cox model after adjusting for age, pre-existing TB, pack-years smoked, and clinical stages.

**TABLE 2:** Stratification Analysis of the CNV-based PRS and NSCLC survival.

Variables	Low PRS			High PRS			HR (95%CI) High vs Low	*P_*inter*_^*a*^*
	*n*	Death (%)	MST (month)	*n*	Death (%)	MST (month)		
**Age (years)**								
<55	223	149 (66.8)	18	82	66 (80.5)	13	1.53(1.14–2.05)	0.991
55–70	318	224 (70.4)	15	131	99 (75.6)	13	1.29(1.02–1.64)	
>70	194	150 (77.3)	9	87	80 (92.0)	7	1.43(1.09–1.88)	
**Pre-existing TB**								
No	682	484 (71.0)	15	270	218 (80.7)	11	1.41(1.20–1.66)	0.782
Yes	53	39 (73.6)	11	30	27 (90.0)	8	1.28(0.78–2.10)	
**Pack-year smoked**								
<5	510	352 (69.0)	16	211	172 (81.5)	12	1.44(1.20–1.73)	0.715
5–20	54	37 (68.5)	13	25	20 (80.0)	5	1.41(0.79–2.52)	
20–50	126	99 (78.6)	11	40	33 (82.5)	8	1.30(0.87–1.95)	
>50	45	35 (77.8)	10	24	20 (83.3)	6	1.48(0.83–2.63)	
**Asbestos exposure**								
No	660	475 (72.0)	15	259	209 (80.7)	11	1.34(1.14–1.58)	0.042
Yes	75	48 (64.0)	17	41	36 (87.8)	4	2.04(1.32–3.17)	

**^*a*^*A multiplicative interaction model was supplied for the interaction analysis.*

## Discussion

In this study, we sought to identify gCNVs that affect NSCLC OS. By analyzing the TCGA data and assessing the promising gCNVs in two cohorts of NSCLC patients in southern Chinese, we revealed two gCNVs that are CNVR395.1 and CNVR2239.1 were associated with OS of NSCLC in Chinese. Patients with high PRS, which is calculated by the risk effects of CNVR395.1 and CNVR2239.1, displayed significantly poorer survival than those with low PRS. We also found age, pre-existing TB, pack-years smoked, and asbestos exposure harbored a detrimental contribution to NSCLC OS. The nomogram incorporating these OS-related factors, achieved an admissible concordance index in predicting OS, and had well-fitted calibration curves. In additional analyses assessing gene-environment interaction, we found that asbestos exposure interacted with PRS on affecting NSCLC OS. To our knowledge, this study is among the few studies to explore OS-related gCNVs at a genome-wide level for NSCLC.

Several gCNVs have been studied as genetic determinants for prognosis of cancers, including breast (Sapkota et al., 2013; Kumaran et al., 2017), colorectal (Andersen et al., 2011; Werdyani et al., 2017), ovarian (Fridley et al., 2012), and prostate (Jin et al., 2011). In contrast, relevance of gCNVs to NSCLC prognosis is largely unknown. We have previously identified two gCNVs namely CNV-30450 in *MAPKAPK2* and CNV-3956 in *CHRNA7* as prognostic biomarkers for lung cancer survival based on candidate gene study design (Liu et al., 2012; Yang et al., 2015b). Here, at the genome-wide level, we identified CNVR395.1 and CNVR2239.1. It is well known that CNVs affect human diseases *via* modulatory effects on embedded genes (Yang et al., 2013, 2018; Fehrmann et al., 2015). Being recorded as dgv624n67 in the Database for Genomic Variants (DGV^4^), CNVR395.1 is located on the chromosome 2p21 (hg38: chr2:41548788-41550118), a region where driver mutations were identified for lung cancer (Dano et al., 2000). Since CNVR395.1 maps to the first intron of an uncharacterized long non-coding RNA that is LOC105374506, it is really difficult to evaluate the biological function of the gCNV. Otherwise, it may be just a genetic biomarker. As reported (Park et al., 2010), CNVR2239.1 is located on the chromosome 10p12.31 (hg38: chr10:20549685- 20550930), which corresponds to dgv132n67 in the DGV database. Our AGE test demonstrated the veritable region of the gCNV is much larger than that as reported. Thus, CNVR2239.1 may completely encompass the whole DNA sequences of MIR4675 instead of just covering the promoter region. MIR4675 had been reported to be a susceptible microRNA for eosinophilic esophagitis risk (Kottyan et al., 2014). A putatively biological role of CNVR2239.1 is that the copy changes induce a gene dosage effect on MIR4675 expression. Further works are warranted to establish the role of MIR4675 and CNVR2239.1 in lung cancer.

Although many studies have reported nomograms for estimating survival rate of patients affected by NSCLC (Liang et al., 2015; Tian et al., 2020; Vaidya et al., 2020), constructing a novel nomogram based on gCNVs and surrounding factors is still scientific and innovative. The phenotype of gCNV is constant over time and easy to detect. Meanwhile, it is quite convenient to collect the information on surrounding factors. Thus, using this model to evaluate OS of patients with NSCLC will be extremely easy to implement.

So far there was no study investigating the gCNV-environment interaction on lung cancer survival. We for the first time observed a positively significant gene–environment interaction between the gCNVs-based PRS and asbestos exposure on worsening NSCLC OS. Asbestos cement workers exert higher lung cancer mortality than general population (Cuccaro et al., 2019). Also, asbestos exposure increases lung cancer risk (Klebe et al., 2019; Brims et al., 2020). Being consistent, we found that asbestos exposure also conferred a poor survival to NSCLC patients. This data suggested that the gCNVs could enlarge the adverse effect of asbestos exposure on lung cancer OS.

Our study had limitations. First, we lacked data on the disease-free survival (DFS), which is also an important feature for cancer patients. Second, we lacked the treatment information, which might bias the correlation between all prognostic factors and NSCLC OS.

In summary, our findings lead to an understanding of gCNVs’ contribution to lung cancer OS and suggested that CNVR395.1 and CNVR2239.1 affect the carriers’ survival *via* interaction with asbestos exposure. The nomogram incorporating the gCNVs and surrounding factors achieved an admissible prediction of NSCLC survival, which would be beneficial to personalized intervention in future.

## Data Availability Statement

The raw data supporting the conclusions of this article will be made available by the authors, without undue reservation.

## Ethics Statement

The studies involving human participants were reviewed and approved by the institutional review boards of Guangzhou Medical University and Soochow University. The patients/participants provided their written informed consent to participate in this study.

## Author Contributions

LY conceived and designed the study with support from JL and FQ. LY wrote the first draft of the manuscript. SC and CS wrotethe R code and performed statistical analysis. SC, LL, and JX carried out the experiments. JC and BR contributed to sample preparation. All authors interpreted the results, revised and approved the manuscript for submission.

## Conflict of Interest

The authors declare that the research was conducted in the absence of any commercial or financial relationships that could be construed as a potential conflict of interest.

## Footnotes

1 https://gco.iarc.fr/today/home

2 https://assets.thermofisher.com/TFS-Assets/LSG/manuals/4397425_CopyNumAssays_UG.pdf

3 https://tools.thermofisher.com/content/sfs/manuals/cms_062369.pdf

4 http://dgv.tcag.ca/dgv/app/home

## Supplementary Material

Supplementary Figure 1 Genotyping of copy number of CNVR395.1 and CNVR2239.1. **(A)** The Accucopy assay was performed to determine the copy number of CNVR395.1 (left) and CNVR2239.1 (right). **(B)** The Taqman copy number assay was performed to determine the copy number of CNVR395.1 (left) and CNVR2239.1 (right).

Supplementary Figure 2 Agarose gel electrophoresis assay determining the existence of CNVR395.1 and CNVR2239.1. **(A)** Schematic representation of CNVR395.1 and PCR-amplified region of DNA with designed primers (Upper), and gel electrophoresis image for PCR bands (Below). **(B)** Schematic representation of CNVR2239.1 and PCR-amplified region of DNA with designed primers (Upper) and gel electrophoresis image for PCR bands (Below).
