# Supplementary material for: Prognostic Value of Germline Copy Number Variants and Environmental Exposures in Non-small Cell Lung Cancer
Source: Front Genet. 2021 Jun 11;12:681857. doi: 10.3389/fgene.2021.681857 (PMC8226327; doi:10.3389/fgene.2021.681857)
Supplement: Supplementary file 2 [file Table_1.docx]

**Supplementary Table 1.** Primer information used for Agarose gel electrophoresis test.

| Target CNV | Target fragment | Sequence information  5' −> 3' | Product  size (bp) |
| --- | --- | --- | --- |
| CNVR395.1 | P1 | catccccaggatgcaaggttgg(Forward) | 140 |
|  |  | tgaatgggcatcaaattccgttga(Reserve) |  |
|  | P2 | TCTTGCCCACCCTCACAAATA(Forward) | 505 |
|  |  | GTGAAGATGAGCAGATTCACAG(Reserve) |  |
|  | P3 | gtgctcctgtattgtccctgtt(Forward) | 504 |
|  |  | ggattgacttggcgatgc(Reserve) |  |
|  | P4 | cttgcccaccctcacaaataaaat(Reserve) | 313 |
|  |  | ttgtgatgatgaagatgtgccaga(Forward) |  |
|  | P5 | catcagaagcagctgcaagggtaagca(Reserve) | 386 |
|  |  | ttggcctttcaacagtgggggttg(Forward) |  |
|  |  |  |  |
| CNVR2239.1 | P6 | AATGAGAGCCTCACCAAAGAGC(Forward) | 196 |
|  |  | GCCCAGCTTCTGATGTTCAC(Reserve) |  |
|  | P7 | GATGGGCGTATGATGGGGTT(Forward) | 198 |
|  |  | CCCTCAGTATACCCCTATTACATC(Reserve) |  |
|  | P8 | TTGCAGTCACAGCTACCGAG(Forward) | 195 |
|  |  | AGGTAGGGGTGTCTCACAAT(Reserve) |  |
