# Supplementary material for: Prognostic Value of Germline Copy Number Variants and Environmental Exposures in Non-small Cell Lung Cancer
Source: Front Genet. 2021 Jun 11;12:681857. doi: 10.3389/fgene.2021.681857 (PMC8226327; doi:10.3389/fgene.2021.681857)
Supplement: Supplementary file 3 [file Table_2.docx]

**Supplementary Table 2.** List of survival-related gCNVs with *P* < 0.01 from the Cox analysis on TCGA NSCLC patients.

| CNV ID | Chromosome location | Cox test *P* value | CNV ID | Chromosome location | Cox test *P* value |
| --- | --- | --- | --- | --- | --- |
| CNVR_563.1 | 2:178564414-178565438 | 7.76×10^-06^ | CNVR_1837.1 | 8:5715393-5716952 | 0.003657398 |
| CNVR_1999.1 | 9:1698103-1699003 | 2.06×10^-05^ | CNVR_2748.1 | 12:34308821-34447583 | 0.004244808 |
| CNVR_642.1 | 3:3455975-3457322 | 5.00×10^-05^ | CNVR_2011.1 | 9:6000950-6004414 | 0.00475604 |
| CNVR_1956.1 | 8:131919840-131922045 | 6.73×10^-05^ | CNVR_1635.1 | 7:22401248-22403322 | 0.004799569 |
| CNVR_1406.1 | 6:22158952-22162332 | 0.00013986 | CNVR_3403.1 | 16:76929024-76942626 | 0.005273892 |
| CNVR_1986.1 | 8:144987655-144988995 | 0.000705882 | CNVR_336.1 | 1:246794322-246863301 | 0.005371061 |
| CNVR_3560.1 | 17:53042759-53044903 | 0.000849836 | CNVR_2186.1 | 9:137353872-137357550 | 0.007180815 |
| CNVR_3262.1 | 15:96659788-96661543 | 0.00121826 | CNVR_560.1 | 2:176630166-176789197 | 0.007672403 |
| CNVR_1070.1 | 4:142450005-142452400 | 0.00130144 | CNVR_561.1 | 2:176973832-176980546 | 0.007672403 |
| CNVR_431.1 | 2:66359055-66361142 | 0.001413732 | CNVR_2239.1 | 10:20878620-20879865 | 0.008259664 |
| CNVR_1765.1 | 7:133435616-133448477 | 0.002045436 | CNVR_1713.1 | 7:90870713-90879046 | 0.008469716 |
| CNVR_1765.2 | 7:133441968-133443252 | 0.002045436 | CNVR_6.1 | 1:2227388-2231840 | 0.008914695 |
| CNVR_3105.1 | 14:98572986-98575243 | 0.002282555 | CNVR_1833.1 | 8:5168864-5169401 | 0.009131375 |
| CNVR_645.1 | 3:5510233-5514308 | 0.002637353 | CNVR_395.1 | 2:41629432-41630762 | 0.009444727 |
| CNVR_2185.1 | 9:137333761-137335583 | 0.00274824 | CNVR_564.1 | 2:179004316-179005350 | 0.009581044 |
| CNVR_1675.1 | 7:56729530-56735824 | 0.002979536 | CNVR_2703.1 | 12:7846734-7847884 | 0.00989322 |
| CNVR_1866.1 | 8:24201155-24207060 | 0.003376289 | CNVR_3034.1 | 14:34675160-34684010 | 0.009982389 |
| CNVR_1447.1 | 6:51307388-51308060 | 0.003555994 |  |  |  |
