# Supplementary material for: Prognostic Value of Germline Copy Number Variants and Environmental Exposures in Non-small Cell Lung Cancer
Source: Front Genet. 2021 Jun 11;12:681857. doi: 10.3389/fgene.2021.681857 (PMC8226327; doi:10.3389/fgene.2021.681857)
Supplement: Supplementary file 4 [file Table_3.docx]

**Supplementary Table 3.** Analysis of associations between 15 potential predictors and OS of NSCLC patients in southern Chinese.

| CNV ID | Cox test *P* value | CNV ID | Cox test *P* value | CNV ID | Cox test *P* value |
| --- | --- | --- | --- | --- | --- |
| CNVR_563.1 | 0.263118337 | CNVR_2185.1 | 0.599480017 | CNVR_560.1 | 0.821371861 |
| CNVR_642.1 | 0.971000365 | CNVR_1866.1 | 0.301943287 | CNVR_2239.1 | 0.023633222 |
| CNVR_1956.1 | 0.156140617 | CNVR_1837.1 | 0.923593616 | CNVR_395.1 | 0.00119848 |
| CNVR_3560.1 | 0.366525562 | CNVR_2748.1 | 0.693293218 | CNVR_564.1 | 0.97807646 |
| CNVR_431.1 | 0.966810679 | CNVR_2186.1 | 0.152917686 | CNVR_2703.1 | 0.129620806 |
