# Supplementary material for: Prognostic Value of Germline Copy Number Variants and Environmental Exposures in Non-small Cell Lung Cancer
Source: Front Genet. 2021 Jun 11;12:681857. doi: 10.3389/fgene.2021.681857 (PMC8226327; doi:10.3389/fgene.2021.681857)
Supplement: Supplementary file 5 [file Table_4.docx]

**Supplementary Table 4.** Multivariate Cox Regression Analysis of environmental exposures, PRS and NSCLC survival.

| Factors | HR (95%CI) | Cox model *P* value |
| --- | --- | --- |
| Age (years) | 1.26 (1.08-1.47) | 0.003 |
| Pre-existing TB | 1.28 (0.98-1.67) | 0.070 |
| Pack-year smoked | 1.15 (1.05-1.25) | 0.002 |
| Asbestos exposure | 1.23 (0.95-1.60) | 0.120 |
| Clinical stages | 1.52 (1.40-1.64) | <0.001 |
| PRS | 1.48 (1.26-1.75) | <0.001 |
