# Supplementary material for: Prognostic Value of Germline Copy Number Variants and Environmental Exposures in Non-small Cell Lung Cancer
Source: Front Genet. 2021 Jun 11;12:681857. doi: 10.3389/fgene.2021.681857 (PMC8226327; doi:10.3389/fgene.2021.681857)
Supplement: Supplementary file 6 [file Data_Sheet_2.pdf]

1.1 R code used for recompiling the copy number data from TCGA into identifiable CNVs for East Asian:

```
rm(list=ls())
asian = read.table("C:\\....\\asian_1.txt",header=T)    # database file of identifiable CNVs for East Asian. The file was downloaded from the Park's paper (Park and others 2010) as record as Supplementary Table S7 (https://www.ncbi.nlm.nih.gov/pmc/articles/PMC3329635/)
sample_dir =dir("C:\\...\\samples")     # file for all samples including CNV data of NSCLC patients in TCGA database. Each individual has one file. An example can be found as attached in this paper.
file = paste("C:\\...\\samples\\",sample_dir,sep="")
totalCNV = c()
for (l in 1:length(file)){
sample = read.table(file[l],header=T) # sample data
sample_cnv = 0
for (i in 1:length(asian$start)){
   print(i)
   sample01 = sample[sample$Chromosome==asian$chr[i],]
   for(j in 1:length(sample01$Start)){
      if((asian$start[i]>=sample01$Start[j])&(asian$start[i]<=sample01$End[j])
         ){
          sample_cnv[i]=sample01$CNV[j]
         break
      }
      if( is.na(sample_cnv[i])){
         for(k in 1:length(sample01$End)){
         if((asian$stop[i]>=sample01$Start[k])&(asian$stop[i]<=sample01$End[k])
              ){
            sample_cnv[i]=sample01$CNV[k]
            break
```

```
            }
            }
        }
        }
}
totalCNV = cbind(totalCNV,sample_cnv)
}
```

# Match the CNV information according to their chromosomal location.

```
total = data.frame(CNVE=asian$CNVE,totalCNV)
colnames(total) = c('CNVE',t(sub('....$','',sample_dir)))
write.table(total, file = "D:\\csv_data\\result.txt", row.names=F, quote=F)
```

# export the data.
