# Supplementary figures and images for: Prognostic Value of Germline Copy Number Variants and Environmental Exposures in Non-small Cell Lung Cancer
Source: Front Genet. 2021 Jun 11;12:681857. doi: 10.3389/fgene.2021.681857 (PMC8226327; doi:10.3389/fgene.2021.681857)

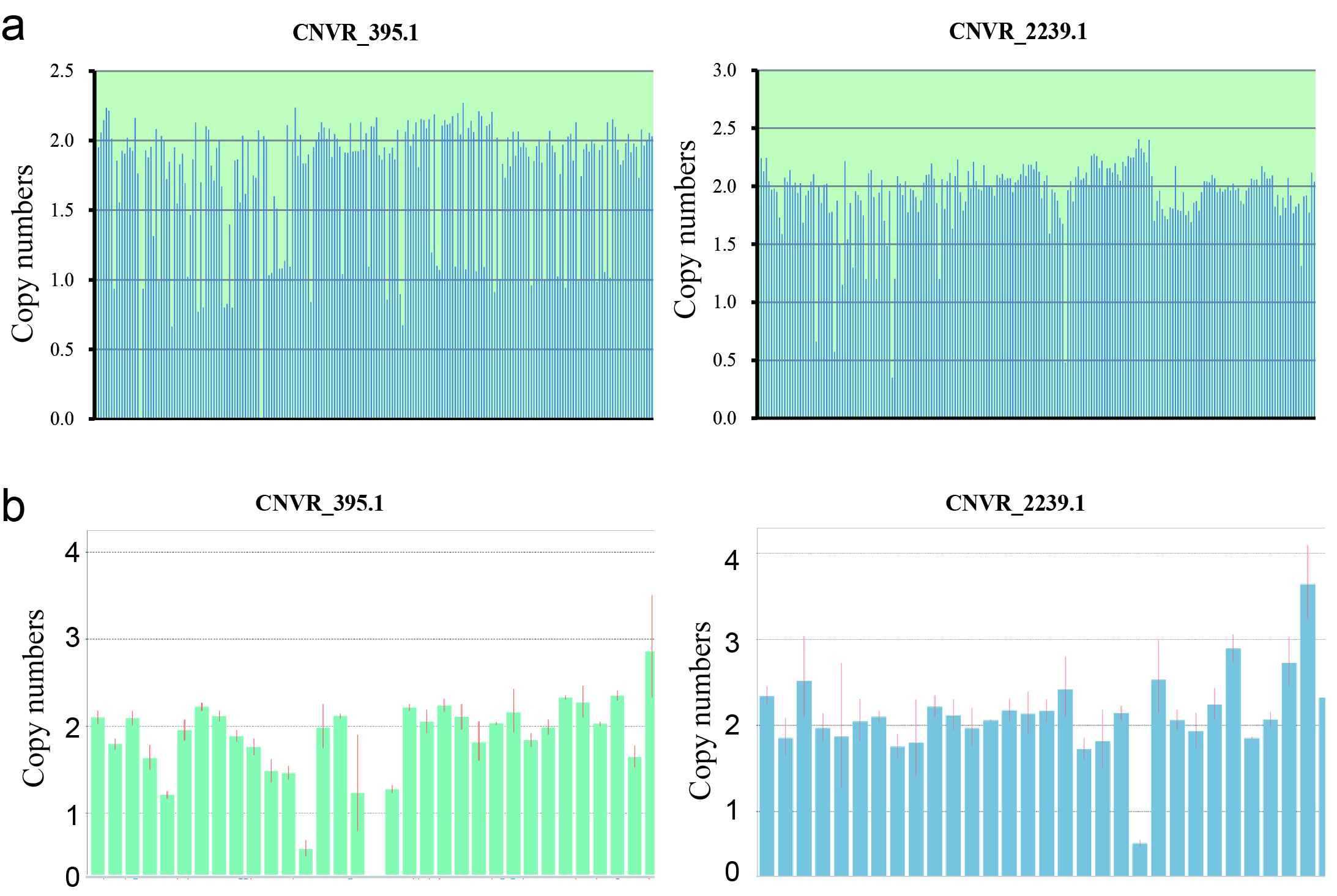

Supplement: Supplementary Figure 1 — Genotyping of copy number of CNVR395.1 and CNVR2239.1. (A) The Accucopy assay was performed to determine the copy number of CNVR395.1 (left) and CNVR2239.1 (right). (B) The Taqman copy number assay was performed to determine the copy number of CNVR395.1 (left) and CNVR2239.1 (right). [file Image_1.JPEG]

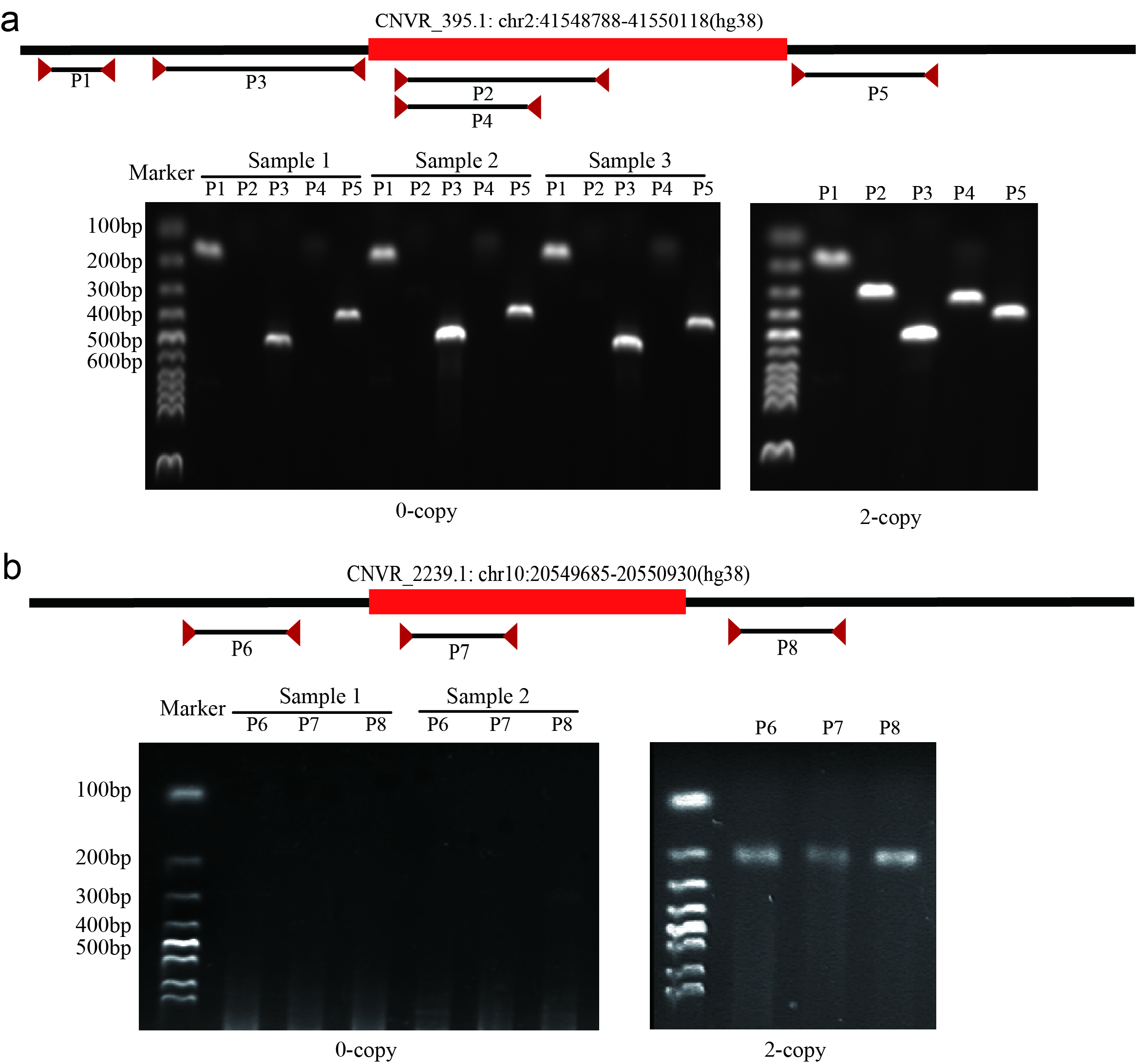

Supplement: Supplementary Figure 2 — Agarose gel electrophoresis assay determining the existence of CNVR395.1 and CNVR2239.1. (A) Schematic representation of CNVR395.1 and PCR-amplified region of DNA with designed primers (Upper), and gel electrophoresis image for PCR bands (Below). (B) Schematic representation of CNVR2239.1 and PCR-amplified region of DNA with designed primers (Upper) and gel electrophoresis image for PCR bands (Below). [file Image_2.JPEG]
